# Genetics, morphology and diet of introduced populations of the ant-eating Texas Horned Lizard (*Phrynosoma cornutum*)

**DOI:** 10.1038/s41598-019-47856-4

**Published:** 2019-08-07

**Authors:** Courtney Heuring, Diane Barber, Nathan Rains, Devin Erxleben, Cameron Martin, Dean Williams, Eric J. McElroy

**Affiliations:** 1 Department of Biology, College of Charleston, Charleston, South Carolina 29412 USA; 2 Fort Worth Zoo, Fort Worth, Texas 76110 USA; 3 Texas Parks and Wildlife Department, Austin, Texas 78744 USA; 4 Department of Biology, Texas Christian University, Fort Worth, Texas 76129 USA

**Keywords:** Herpetology, Ecological genetics, Invasive species

## Abstract

Introduced species can diverge from their source population when they become established in a new ecosystem. The Texas Horned Lizard (*Phrynosoma cornutum*) is native to the western United States (US) and was historically introduced to several locations in the southeastern US. We studied three introduced populations in South Carolina, US to determine if they exhibit dietary, morphological and genetic divergence from the native western US populations. We expected little divergence from western populations because *P. cornutum* is a specialist whose biology is largely shaped by its diet of *Pogonomyrmex* harvester ants. We show that the introduced populations have mixed ancestry between south Texas and more northern areas and experienced founder effects and genetic bottlenecks resulting in decreased genetic diversity. South Carolina lizards primarily consume ants (94%), but surprisingly, they did not eat harvester ants. Introduced lizards primarily eat *Dorymyrmex* ants, but each introduced population complements *Dorymyrmex* with significantly different amounts of other species of ants, insects and plant matter. Introduced populations have smaller body size and have different limb and head shapes compared to western populations. This study demonstrates successful persistence of an introduced vertebrate that may be attributed to phenotypic change, even in the face of reduced genetic diversity.

## Introduction

The number of non-native species is estimated at >15,000 worldwide and the rate of invasion is increasing^1^. The main concern with non-native species is that they are often invasive, which occurs when they have detrimental impacts on native species and ecosystem processes^2^. However, not all introductions are detrimental, with some non-native species causing changes to ecosystem dynamics that benefit native species and others causing little or no effect^3–5^.

Non-native species must effectively deal with suites of environmental and biological factors if they are to become established. Non-native species may find competition, novel predators, and few or different resources in the invaded habitat. These factors can exert selection and this may cause phenotypic and/or genetic changes in the non-native species to enable their persistence^6–8^. This can result in the non-native species effectively competing for a limiting resource or it could result in a shift to a novel resource^9,10^. For example, morphological changes often occur after invasion of a new area^11–14^. Collectively, these changes may result in increased fitness and rapid population growth^15^.

Conversely, non-native species may already have phenotypes well-suited for the invaded area; they are pre-adapted^6,16^. The invaded area may have a resource pool similar to the source population, there may be few competitors for those resources, predators may not target the non-native species and environmental factors may be similar^16–18^. This may result in very little phenotypic or genetic change in the non-native species when compared to the source population^19^. Quantifying potential changes in non-native species compared to its own native range is important because it could give insight into their impact on native ecosystems.

Although selection and phenotypic change are clearly important in determining the success of many non-native species; genetic bottlenecks and founder effects reduce genetic variation which can slow the response to selection^6,20^. However, while genetic variation is often decreased after invasion, quantitative trait variation is rarely altered^20^. Decoupling genetic and quantitative trait variation occurs for several reasons. First, quantitative traits are affected by many genes and are often robust to loss of rare alleles^21^. Second, loss of genetic variation is sometimes recovered because dominance and epistatic variance may be converted to additive genetic variance during the bottleneck; this can be especially high for founder events with few individuals^22–24^. In addition, mutations can rapidly accumulate and impact quantitative traits, especially in species with rapid, post-introduction population growth^21^. Third, the molecular markers used to quantify genetic variation are neutral and may have little or no direct impact on quantitative phenotypic traits^25–27^. Thus, loss of total genetic variation might be coupled with a stable or increased additive genetic variation and maintenance of quantitative trait variation after a bottleneck. Although reduction of genetic variation may not impact quantitative trait variation, it often results in inbreeding depression and reduced fitness. When coupled with small population size and new selective pressures, reduced genetic variation might limit that ability of many non-native species to persist^28^.

Barrier islands are elongate landmasses that are parallel to the shoreline and separated from the mainland by bays or lagoons and from each other by inlets; they are a major geographical feature of the coastline of the southeastern United States^29^. Barrier island ecosystems provide unique habitats that support a variety of species. However, they also have been greatly impacted by humans and are prone to flooding and wind-associated damage caused by tropical cyclones^30,31^. These factors collectively cause disturbance to barrier island ecosystems which may encourage the establishment of non-native species^32^. In the southeastern United States, the fire ant (*Solenopsis invicta*) and beach vitex (*Vitex rotundifolia*) are invasive on barrier islands^33,34^.

The Texas Horned Lizard (*Phrynosoma cornutum*) was introduced in the Carolinas and Florida^35–37^. This species was first introduced to these areas during the 1920’s–1940’s and was thought to be released as a type of pest control in Florida and as released ‘pets’ by soldiers stationed at military bases in the Carolinas^38,39^. Since the introduction, *P. cornutum* has been reported in several locations along the east coast including Sullivan’s Island, Isle of Palms, Edisto Island, and Murrells Inlet in South Carolina^40^. The native range of *P. cornutum* extends from Kansas and Colorado at the north, through Oklahoma, Texas, New Mexico, and parts of Arizona into northern Mexico^37^. It is unknown if the populations in the southeastern US represent a single introduction and subsequent spread, multiple introductions or some combination of both. Discovering the source population(s) is particularly important for making comparisons between native and introduced ranges^41^.

The persistence of *P. cornutum* in the southeastern US is somewhat of an enigma because horned lizards are generally myrmecophagous with many species specializing on harvester ants^42^. In particular, western populations of *Phrynosoma cornutum* primarily consume *Pogonomyrmex* harvester ants^37,43–47^. Recently, *P. cornutum* has disappeared in many of the southwest locations where it was historically abundant, and this decline is thought to be due to food shortage and habitat loss^37,48,49^. In particular, loss of *Pogonmyrmex* ants associated with fire ant invasion and pesticide use is hypothesized as a primary driver of Texas Horned Lizard declines^44,50^. In the eastern United States, the Florida harvester ant (*Pogonomyrmex badius*) inhabits the sandy dune habitats and is the only harvester ant present^51^ and thus may represent a food source that has sustained introduced populations of *P. cornutum*.

In this study we confirm the presence of *P. cornutum* populations at three different South Carolina (SC) locations and compare them to each other and to the native populations in the western United States. Specifically, we test the following hypotheses (1) SC populations represent a subset of genetic variation relative to Texas, (2) SC populations were established from few (versus multiple) introduction events, (3) SC populations exhibit no differences among each other or compared to western populations in the body size, limb shape or head shape, and (4) the diet of SC lizards is composed of at least 51% *Pogonomyrmex* harvester ants by number, similar to the diet of western populations. We expect little differentiation in morphology and ecology because *P. cornutum* is a highly specialized myrmecophage and a terrestrial habitat generalist.

## Methods

### Study sites and sampling

We hand-captured *P. cornutum* from May 2014 – July 2015 at three study sites in southeastern, coastal South Carolina (Isle of Palms [IOP; N = 62], Sullivan’s Island [SI; N = 40], and Edisto Beach [E; N = 26]). We marked them via a unique toe-clip pattern, and later released them at the GPS coordinates of capture. We also captured additional lizards at Chaparral Wildlife Management Area (WMA) in southern Texas (N = 5) and Irion and Crockett counties in western Texas (N = 21). Lizards captured in Texas were only used for the morphological analysis (see below).

### Population genetics

Texas Horned Lizard tissue samples (cloacal swabs or toe clips, N = 741 individuals) were obtained in Texas, New Mexico, Colorado, and Oklahoma between 2009–2017 by a large number of volunteers and from other research projects. Additionally, we obtained tissue samples from a total of 91 horned lizards in SC in Isle of Palms (N = 37), Sullivan’s Island (N = 29), and Edisto Beach (N = 25). DNA was extracted from samples and genotyped at 10 microsatellite loci^52^. We also amplified a 353 bp section of the mitochondrial control region (d-loop) using the primers PcCR_F: 5′-CTTATGATGGCGGGTTGCT-3′ and PcCR_R: 5′-GGCTGTTAAATTTATCCTCTGGTG-3′. Polymerase chain reactions (PCR) (10 µL) contained 10–50 ng DNA, 0.5 µM of each primer, 1X Qiagen Multiplex PCR Master Mix with HotStarTaq, Multiplex PCR buffer with 3 mM MgCl_2_ pH8.7, and dNTPs. Reactions were cycled in an ABI 2720 thermal cycler. The cycling parameters were one cycle at 95 °C for 15 min, followed by 30 cycles of 30 s at 94 °C, 90 s at 55 °C, 90 s at 72 °C, and then a final extension at 72 °C for 5 minutes. We sequenced products using ABI Big Dye Terminator Cycle Sequencing v3.1 Chemistry (Life Technologies) using the PCR primers. Sequences were electrophoresed on an ABI 3130XL Genetic Analyzer (Life Technologies); edited and trimmed using Sequencher v5.0 (Gene Codes USA); and then aligned in MEGA 6.0^53^ using Muscle^54^. We identified haplotypes using GenAlEx v6.5^55^.

### Morphology

We measured, massed, and sexed all lizards captured in SC and TX. We used digital calipers to take the following 13 measurements: body (snout-vent length and tail length), fore limb (brachium, antebrachium, wrist, and longest fore toe length), hind limb (thigh, shank, foot, and longest hind toe length) and head (width, height, and jaw length). We took all measurements on the lizard’s right side unless there was damage, in which case we measured the left side. We chose these specific measurements because they are the morphological traits commonly studied in *Phrynosoma* and can be impacted by environmental variation^56–58^. Five individuals were caught as a juvenile and then recaptured as an adult in the next season; we counted these as separate data points. We excluded 10 individuals from morphological analysis because they had broken tails.

### Dietary analysis

We collected fresh *P. cornutum* fecal pellets in the field (easily recognized by unique cylindrical shape of the feces) from each of the three SC study sites and preserved them in 95% ethanol. We analyzed ten fecal pellets from each of the SC sites for a total of 30 samples. In addition, we obtained stomach contents via gastric lavage from nine Isle of Palms lizards^59^. Additionally, we obtained seven historical *P. cornutum* specimens from the University of Michigan Museum of Zoology (UMMZ) that originated in the Charleston, SC area from the years 1954–1982. We removed the gut contents from these historical specimens. We dissected all samples and visually identified prey items to genus for ants and order for other insects.

### Statistical analyses

#### Microsatellite data

We compared the SC populations to five populations from large natural protected areas in Texas that were analyzed in Williams *et al*. (2019): Matador WMA (wildlife management area) (N = 55 individuals), Chaparral WMA (N = 63), Yoakum Dunes WMA (N = 36), Rolling Plains Quail Research Ranch (RPQRR) (N = 79), Seminole Canyon State Park (N = 17), and Matagorda Island WMA (N = 30) in Texas. Previous analysis revealed that the Matagorda Island WMA barrier island population has lower genetic diversity than the mainland areas suggesting they have been bottlenecked^60^. We used GENEPOP v4.5 to test for Hardy-Weinberg and genotypic linkage equilibrium^61^. We used sequential Bonferroni correction to determine significance for these tests. MICRO-CHECKER 2.2.3^62^ was used to determine the presence of null alleles, large allele dropout, or issues with scoring due to stuttering. As there was some evidence of null alleles, we used the ENA correction method^63^ implemented in the software FreeNA to calculate F_ST_. We calculated the number of alleles, observed heterozygosity (H_o_), expected heterozygosity (H_e_), the inbreeding coefficient (F),using GenAlEx v6.5^55^. We calculated allelic richness using HP-Rare to control for sample size differences between the populations^64^. We estimated the effective population sizes using the linkage disequilibrium method in NeEstimator v2.01^65^. The lowest allele frequency used was 0.01 and the 95% confidence intervals were estimated using the jack-knife method.

We used the software STRUCTURE v2.3^66^ to assign South Carolina samples to previously defined genetic regions in Williams *et al*. (2019). Previous analyses have found two major mitochondrial clades and three major genetic groupings at nuclear microsatellite loci, a western group corresponding with the western mitochondrial clade and a northern and southern group within the eastern mitochondrial clade^60^. We chose a subset of individuals from that study (N = 484) that had high ancestry (q > 0.90) in each of the three microsatellite genetic groups (northern, western, southern) and used them as training samples to assign the SC samples to a particular region. We assumed admixture, correlated allele frequencies, updated the allele frequencies using only the training samples, and allowed ALPHA to vary between clusters. We used a burn-in of 50,000 iterations and ran the MCMC (Monte Carlo Markov Chain for 10^6^ iterations for 10 independent runs at K = 3. We used the software CLUMPP^67^ to estimate average cluster membership across replicates for K.

#### Mitochondrial d-loop data

We used GenAlEx v6.5 to estimate the haplotype diversity and compared haplotypes to previously described haplotypes^60^.

#### Morphology

We tested for population differences in body size using two different methods. First, we computed the geometric mean of the 13 morphological measurements and this value was used as an estimate of size^68^. The advantage of the geometric mean approach is that it computes ‘size’ based on all morphological measurements. Second, we used snout-vent length as a proxy for size, as this measurement is more traditionally used by herpetologists to define body size. To test for differences in body size, we used JMP v12.1.0^69^ to run separate one-way ANOVAs (for males, females and juveniles) with log_10_ geometric mean ‘size’ as the response and study site as the main effect. Locations were compared using Tukey HSD tests. An identical analysis was run using log_10_ snout-vent length as the response.

We tested for populations differences in morphological ‘shape’ by first converting all 13 morphological measurements using Mosimann’s method^68^. To do this, we subtracted the log_10_ geometric mean ‘size’ from each of the log_10_ transformed morphological measurements, resulting in 13 morphological ‘shape’ variables^68^. We then used principal components analysis to reduce the dimensionality of the morphological data set, because some populations/sex/age categories had relatively small samples sizes (Supplementary Table 1). Separate principal components analyses were run for females, males and juveniles. Principal component axes with eigenvalues greater than 1 were saved for subsequent analyses (Quinn and Keough 2004). MANCOVA with the saved principal component axes as responses, population as the main effect and log_10_ geometric mean ‘size’ as a covariate was used to test for population differences in ‘shape’. Separate MANCOVAs were run for females, males and juveniles. Within each MANCOVA, planned linear contrasts were constructed that tested the following *a priori* hypotheses: (1) Texas populations differed from South Carolina populations, (2) Edisto differed from Sullivan’s Island, (3) Sullivan’s’ Island differed from Isle of Palms and (4) Isle of Palms differed from Edisto. We plotted the first two canonical axes from the MANCOVA to help visualize how populations were positioned in multivariate morphological shape space and how the principal components axes explained that positioning.

#### Diet

We created species accumulation curves with cumulative number of prey types as the response and sample number as the predictor for fecal pellets from the three SC study sites and stomach contents from Isle of Palms. We also created curves with cumulative proportion of prey individuals sampled (representing each new prey type) as the response and sample number as the predictor. These figures helped us to determine that we had sufficient sampling using 10 fecal pellets.

Using JMP v12.1.0^69^, we performed Kruskal-Wallis tests of each prey type by location for the fecal pellet data. We also performed comparisons with the historical data. Kruskal-Wallis tests were done on the following: (1) historical gut contents from Isle of Palms, present day stomach contents from Isle of Palms, and present-day fecal pellet data from Isle of Palms and (2) historical gut contents from Sullivan’s Island and present-day fecal pellet contents from Sullivan’s Island. We performed post-hoc Steel-Dwass comparisons with sequential Bonferroni corrections. Additionally, we performed a canonical correspondence analysis (CCA) on the fecal pellet data and then ran an ANOVA permutation test on the model using the ‘vegan’ package in R 3.1.3^70,71^.

All methods were approved by the College of Charleston Institutional Animal Care and Use Committee (protocols: 2012–007 and 2015–007) and followed all applicable laws, rules, and regulations of the United States Government and the State of South Carolina.

## Results

### Population genetics

**Figure 1:**
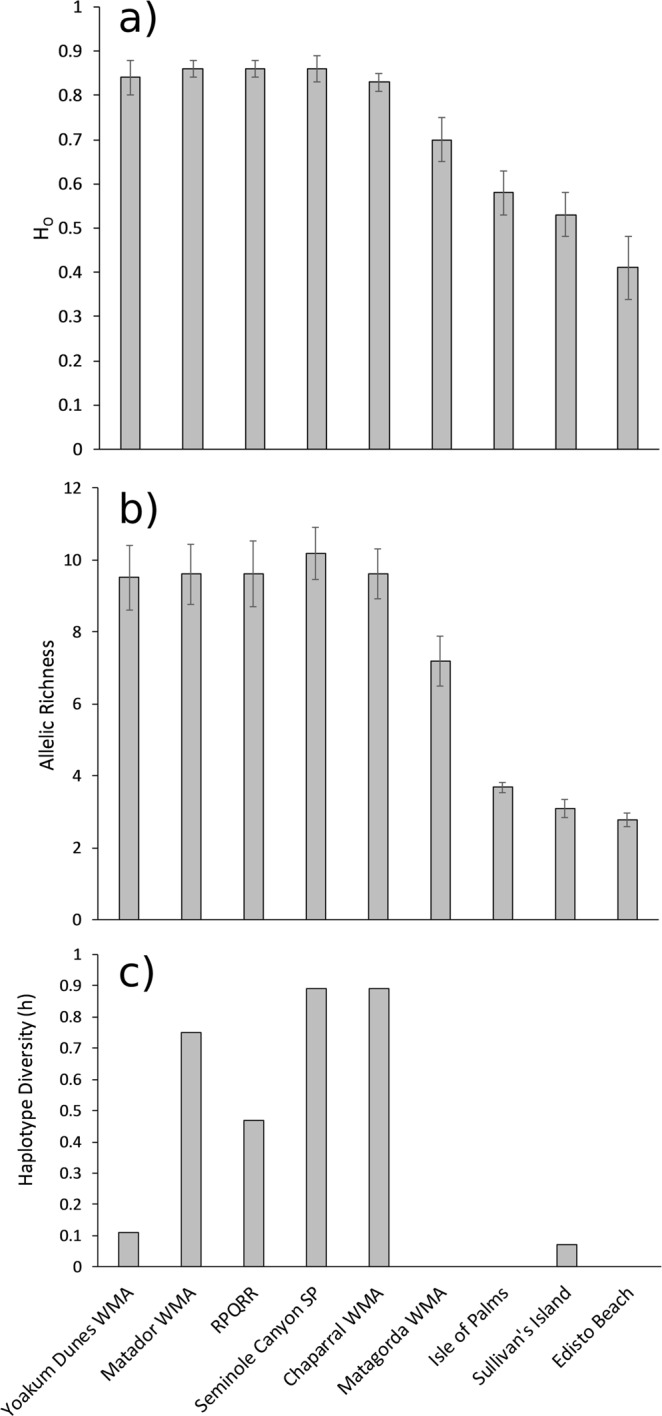
(**a**) Mean (±SE) observed heterozygosity for *Phrynosoma cornutum* populations (N = 10 microsatellite loci). (**b**) Mean (±SE) allelic richness for *P. cornutum* populations standardized to 17 individuals (N = 10 microsatellite loci). (**c**) Haplotype diversity for *P. cornutum* populations.

The three SC populations had low genetic diversity at microsatellite loci with average observed heterozygosity (H_O_) of 0.58 ± 0.05 (SE) at Isle of Palms, 0.53 ± 0.05 at Sullivan’s Island, and 0.41 ± 0.07 at Edisto Beach (Fig. 1a). These values were lower than the heterozygosity observed in the large natural populations in mainland Texas and they were lower than the observed heterozygosity on Matagorda Island WMA (Fig. 1a). All pairs of loci in the SC populations were in genotypic linkage equilibrium except *PcD14* and *PcD53* (P < 0.05) at Isle of Palms. After sequential Bonferroni correction, none of the loci showed heterozygote deficits, but MICRO-CHECKER indicated null alleles were present at two loci *PcD01* and *PcD09* in Isle of Palms (Supplementary Table 2). We reanalyzed these data after removing *PcD01* since this locus also had high F values in the other SC populations but this did not change the results significantly and so we kept all loci in the analyses (data not presented). There was low allelic richness in the SC populations compared to the populations in the west (Fig. 1b). The SC populations were significantly differentiated with an F_ST_ of 0.16 (95% CI: 0.11–0.21). The effective population sizes in the SC populations were 23.5 (95% CI: 8.6–665.1) at Edisto, 26 (95% CI: 16.1–47.3) at Isle of Palms, and 25.2 (95% CI: 11.4–110.5) at Sullivan’s Island which are lower than the point estimate for Matagorda WMA (40.8, 95% CI: 26.1–78.2).

Individuals in the SC populations have a mix of southern and northern ancestry from the native range (Table 1). SC populations had on average 72% southern ancestry and 28% northern ancestry. Both Sullivan’s Island and Isle of Palms had similar levels of southern ancestry (~79%) while Edisto Beach had a higher level of southern ancestry (85%) (Table 1).

**Table 1:** Mean (±SE) ancestry (q) of introduced South Carolina *Phrynosoma cornutum* populations in three native population microsatellite clusters (west, north, south) determined using STRUCTURE.

Location	West	North	South
All	0.01 ± 0.001	0.26 ± 0.02	0.73 ± 0.02
Edisto Beach	0.01 ± 0.005	0.14 ± 0.03	0.85 ± 0.03
Isle of Palms	0.01 ± 0.002	0.31 ± 0.03	0.68 ± 0.03
Sullivan’s Island	0.01 ± 0.001	0.30 ± 0.03	0.70 ± 0.03

One haplotype (H08) occurred in 90 of 91 individuals from all three locations in SC. One individual from Sullivan’s Island had another haplotype (H36). Compared to most of the protected sites in Texas, haplotype diversity in Sullivan’s Island, Isle of Palms, and Edisto Beach was low (h = 0.067, 0, and 0). Matagorda Island WMA also had only one haplotype (Fig. 1c). The common haplotype (H08) found in the SC sites is only found in south Texas in the southern microsatellite genetic cluster and haplotype H36 has not been found in the native range but is most similar to haplotypes H28, H30, and H37. Haplotype H37 is only found in the northern population cluster while H28 and H30 are predominately (79% 59 of 75 individuals) found in the northern cluster but are also found in south Texas (3 individuals) and in west Texas where the western and northern clusters come into contact (13 individuals) (data from^60^). These mitochondrial data are consistent with the results of the microsatellite assignment test which indicated the lizards in SC have a mix of southern and northern ancestry.

### Morphology

**Figure 2:**
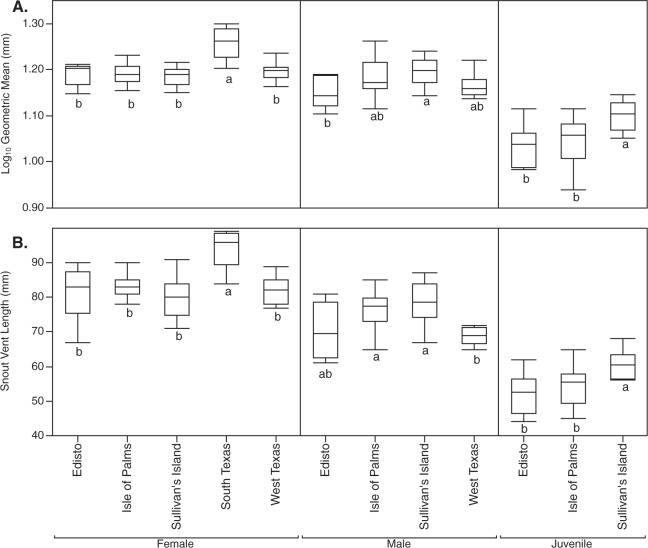
(**A**) Log_10_ geometric mean ‘size’ and (**B**) raw snout-vent lengths (plotted without log_10_ transform) of lizards from different populations. Similar letters within each sex/age/panel category connect populations that do not significantly differ in log_10_ geometric mean ‘size’ or log_10_ snout vent length per Tukey HSD tests. Boxes are the 25th and 75th quartiles, the line within each box are the median and whiskers are the lesser of 1.5* the interquartile range or the upper/lower data values.

Log_10_ geometric mean size significantly differed across populations for females (r^2^ = 0.41, *F*_4,53_ = 9.38, *P* < 0.0001), males (r^2^ = 0.20, *F*_3,46_ = 3.93 *P* = 0.0140) and juveniles (r^2^ = 0.24, *F*_2,33_ = 5.25, *P* = 0.0105). Tukey HSD tests showed several between population differences in geometric mean size (Fig. 2). Log_10_ snout-vent length significantly differed across populations for females (r^2^ = 0.33, *F*_4,53_ = 6.40, *P* = 0.0003), males (r^2^ = 0.25, *F*_3,46_ = 5.05, *P* = 0.0041) and juveniles (r^2^ = 0.24, *F*_2,33_ = 5.15, *P* = 0.0113). Tukey HSD tests showed several between population differences in snout-vent length (Fig. 2).

Principal components analysis on the 13 morphological ‘shape’ variables extracted 5 axes for females, 5 axes for males and 5 axes for juveniles, all with eigenvalues greater than 1 (Table 2). Thus, for each MANCOVA, we used the 5 extracted principal component axes as responses in the next set of analyses.

**Table 2:** Results from principal component analyses on 13 morphological shape variables (adjusted for size, see methods).

	Female					Male					Juvenile				
	PC1	PC2	PC3	PC4	PC5	PC1	PC2	PC3	PC4	PC5	PC1	PC2	PC3	PC4	PC5
Eigenvalue	3.2	2.1	1.8	1.2	1.0	2.9	2.0	1.7	1.4	1.2	3.2	2.5	2.0	1.3	1.0
% Variation	25	16	14	9	8	22	15	13	11	9	25	19	15	10	8
SVL	0.75	0.08	0.40	0.09	−0.03	0.52	−0.50	0.45	0.10	0.19	0.58	0.43	0.05	0.17	0.20
Tail	0.49	0.05	−0.21	−0.49	−0.51	0.32	−0.79	−0.04	0.04	0.02	0.15	0.57	−0.17	−0.17	−0.68
Brachium	0.32	0.58	−0.46	0.22	0.11	0.68	0.41	−0.18	−0.24	−0.22	0.13	0.71	0.30	0.31	0.13
Antebrachium	0.13	−0.28	−0.22	0.77	−0.43	−0.43	0.38	−0.40	0.29	−0.22	0.65	−0.34	−0.10	0.41	0.05
Wrist	−0.63	−0.34	0.40	−0.24	−0.30	−0.50	−0.40	0.14	−0.02	−0.62	−0.32	−0.33	0.74	0.28	0.09
Fore Toe	−0.69	−0.05	−0.29	0.16	0.27	−0.62	0.18	0.23	−0.45	−0.09	−0.41	−0.31	0.49	−0.58	−0.07
Thigh	0.28	0.83	−0.05	−0.18	0.22	0.71	0.25	−0.38	−0.32	−0.08	0.15	0.38	−0.16	−0.45	0.68
Shank	0.03	0.53	0.62	0.25	−0.19	0.21	−0.26	−0.72	0.28	0.24	0.42	0.27	0.51	−0.22	−0.20
Hind Foot	−0.31	−0.08	−0.66	−0.16	−0.00	−0.11	0.34	0.28	−0.35	0.59	−0.66	0.20	−0.36	0.48	−0.05
Hind Toe	−0.54	0.06	0.43	0.13	0.35	−0.63	−0.27	−0.14	−0.00	0.41	−0.77	−0.17	−0.35	−0.02	0.10
Head Width	0.60	−0.37	0.18	0.08	0.24	0.25	0.25	0.38	0.58	−0.09	0.47	−0.08	−0.67	−0.27	−0.03
Head Height	0.56	−0.40	−0.26	0.14	0.22	−0.19	0.42	0.06	0.62	0.18	0.28	−0.79	−0.23	0.01	−0.06
Jaw Length	0.52	−0.55	0.17	−0.20	0.30	0.42	0.18	0.53	0.10	−0.09	0.78	−0.44	0.18	0.07	−0.02

Values presented are the loading between each shape variable and each PC axis.

**Figure 3:**
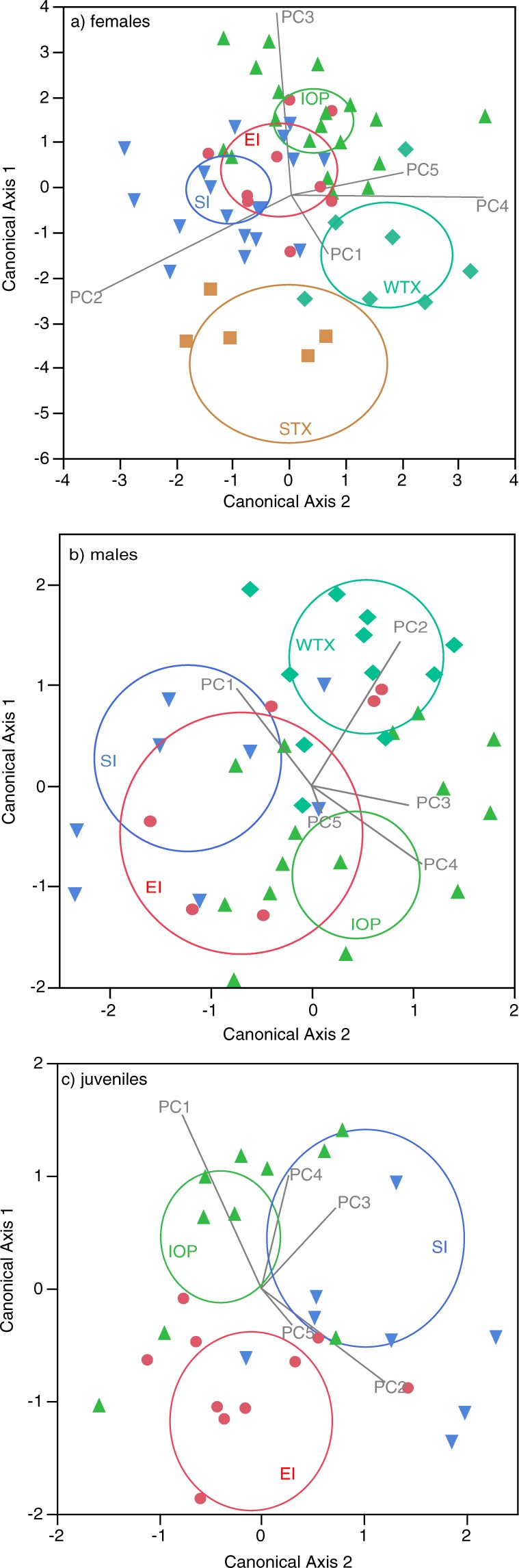
First two canonical axes from MANCOVA on the 13 morphological shape variables with location as a predictor and log_10_ geometric mean ‘size’ as a covariate. Panels are (**a**) females, (**b**) males, and (**c**) juveniles. Symbols as follows: red circles = Edisto Island, green triangles = Isle of Palms, blue inverted triangles = Sullivan’s Island, orange squares = west Texas and teal diamonds = south Texas. The colored ellipses are the 95% confidence intervals for each site. Grey vectors show how each PC axis contributes to the position of individuals in multivariate space.

Female morphological shape significantly differed across locations (MANCOVA: Wilks’ Lambda = 0.127, *F*_20,160.15_ = 6.90, *P* < 0.0001). Females from South Carolina had significantly different morphological shape compared to females from Texas (planned linear contrast: *F*_5,48_ = 14.60, *P* < 0.0001) and this was predominantly due to PC3 (Fig. 3). Thus, South Carolina females had longer shanks, longer hind toes, longer snout-vent lengths and longer wrists but shorter hind feet and shorter brachia when compared to Texas females (Table 2). The three South Carolina populations had significantly different morphological shape when compared to each other (planned linear contrasts: Sullivan’s Island vs. Edisto, *F*_5,48_ = 3.67, *P* = 0.0065; Sullivan’s Island vs. Isle of Palms, *F*_5,48_ = 7.78, *P* < 0.0001; Edisto vs. Isle of Palms, *F*_5,48_ = 5.07, *P* = 0.0008). Differences among females in South Carolina populations was primarily due to PC2 and to a lesser extent PC4 and PC5. PC2 loads positively with thigh, brachium and shank and negatively with jaw length (Table 2); therefore, Sullivan’s Island females have the longest thighs, brachia and shanks and shortest jaws. Edisto females have intermediate thighs, brachia, shanks and jaws. Isle of Palms females have the shortest thighs, brachia and shanks and longest jaws of the South Carolina females.

Male morphological shape significantly differed across locations (MANCOVA: Wilks’ Lambda = 0.328, *F*_15,113.58_ = 3.77, *P* < 0.0001). Males from South Carolina had significantly different morphological shape compared to males from Texas (planned linear contrast: *F*_5,41_ = 6.27, *P* = 0.0002). PC2 was the most important at separating Texas and South Carolina males (Fig. 3). Thus, Texas males had longer brachia, taller heads and shorter snout-vent, tail and wrist lengths compared to South Carolina lizards (Table 2). Among the three South Carolina populations, only Sullivan’s Island had significantly different morphological shape when compared to Isle of Palms (planned linear contrast: *F*_5,41_ = 4.87, *P* = 0.0014). The difference between Sullivan’s Island and Isle of Palms was driven by PC1 and PC4 (Fig. 3). Thus, males from Sullivan’s Island had longer thighs, brachia, snout-vent lengths, and jaws but shorter antebrachia, hind toes, wrists, and shorter, narrower heads when compared to Isle of Palms (Table 2). Edisto males did not differ in morphological shape when compared to Sullivan’s Island males (planned linear contrast: *F*_5,41_ = 0.73, *P* = 0.6090) or when compared to Isle of Palms males (planned linear contrast: *F*_5,41_ = 1.30, *P* = 0.2819).

Juvenile morphological shape significantly differed across locations (MANCOVA: Wilks’ Lambda = 0.498, *F*_10,56_ = 2.34, *P* = 0.0222). Among the three South Carolina populations, only Edisto had significantly different morphological shape when compared to Isle of Palms (planned linear contrast: *F*_5,28_ = 3.09, *P* = 0.0240). The difference between Edisto and Isle of Palms juveniles was driven primarily by PC1 (Fig. 3). Thus, juveniles from Isle of Palms had longer jaws, longer antebrachia, longer snout-vent lengths, longer shanks, wider heads, and shorter fore toes, shorter hind toes, and shorter hind feet when compared to Edisto juveniles. Edisto juveniles did not differ in morphological shape when compared to Sullivan’s Island males (planned linear contrast: *F*_5,28_ = 2.39, *P* = 0.0634). Isle of Palms juveniles did not differ from Sullivan’s Island juveniles (planned linear contrast: *F*_5,28_ = 1.56, *P* = 0.2043).

### Diet composition

A total of 14,847 prey items were found in the 30 fecal pellet samples. Overall, we identified 22 prey types from Isle of Palms, 14 from Sullivan’s Island, and 17 from Edisto Beach. While the species accumulation curves for Sullivan’s Island and Edisto Beach appear to level off, the curve for Isle of Palms did not (Supplementary Fig. 1). Sullivan’s Island had over 97% of the prey individuals represented in just one sample, and Edisto had over 97% represented by two samples. Isle of Palms had 11 prey types found by the third fecal pellet sample and those 11 types represented over 97% of the total prey individuals found in all 10 samples. There was a total of 1187 prey items found constituting 18 prey types found in the stomach contents from Isle of Palms lizards. Over 97% of the prey in the stomach contents were represented by the third sample (Supplementary Fig. 2).

**Figure 4:**
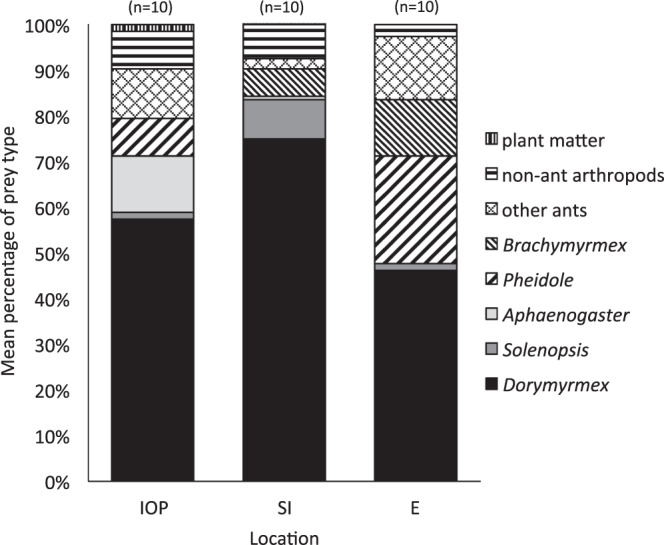
Percentage composition of prey types found in fecal pellets from *Phrynosoma cornutum* at the three SC study sites.

The majority of the diet was composed of ants at all three sites in SC, but Coleoptera, Hemiptera, and plant matter were also found in the fecal pellets (Fig. 4). Ants composed 93.24% of the diet by number with 90.05% at Isle of Palms, 92.43% at Sullivan’s Island, and 97.23% at Edisto. The most common type of prey eaten by lizards was *Dorymyrmex* ants (Fig. 4, Table 3). Some prey types were found only in fecal samples from particular locations, and there were differences among proportions of each type eaten between locations. Sullivan’s Island lizards ate significantly more *Solenopsis* ants than did lizards from Isle of Palms or Edisto (Table 3). Isle of Palms lizards consumed significantly more *Tetramorium* and *Aphaenogaster* ants than did lizards from the other two sites (Table 3). Lizards from Isle of Palms and Edisto consumed significantly more *Forelius* ants than did Sullivan’s Island lizards (Table 3). Lizards from Isle of Palms and Sullivan’s Island occasionally consumed seeds from *Strophostyles* legume plants.

**Table 3:** Diet composition of *P. cornutum* fecal pellets and results from a Kruskal-Wallis Test for diet differences between locations. Mean ± SE are reported.

Prey type	Diet composition (%)			Kruskal-Wallis Results		Group comparisons
	IOP n = 10	SI n = 10	E n = 10	*H*	*P-value*	
Dorymyrmex	57.43 ± 9.55	75.01 ± 5.98	46.05 ± 6.59	6.85	0.0326	SI > E
Camponotus	1.13 ± 0.80	—	0.06 ± 0.06	2.29	0.3177	
Solenopsis	1.20 ± 0.34	8.53 ± 2.72	1.50 ± 1.26	16.33	0.0003*	SI > IOP, SI > E
Tetramorium	4.43 ± 2.03	—	—	11.48	0.0032*	IOP > SI, IOP > E
Aphaenogaster	12.65 ± 4.60	0.51 ± 0.51	—	17.62	0.0001*	IOP > SI, IOP > E
Monomorium	0.23 ± 0.13	—	0.20 ± 0.10	5.11	0.0775	
Forelius	3.36 ± 1.54	0.04 ± 0.04	6.60 ± 3.11	11.97	0.0025*	IOP > SI, E > SI
Pheidole^†^	8.08 ± 5.37	—	23.46 ± 8.84	10.40	0.0055	IOP > SI, E > SI
Paratrechina	0.07 ± 0.07	—	0.01 ± 0.01	1.04	0.5951	
Brachymyrmex	0.13 ± 0.10	6.11 ± 4.07	12.39 ± 6.73	7.84	0.0198	SI > IOP, E > IOP
Crematogaster	—	1.44 ± 1.44	5.97 ± 3.03	5.97	0.0506	
Trachymyrmex	—	—	0.04 ± 0.04	2.00	0.3679	
Other ants^‡^	1.34 ± 0.54	0.79 ± 0.58	0.97 ± 0.47	2.79	0.2473	
Coleoptera	0.84 ± 0.20	4.53 ± 2.83	1.86 ± 1.41	4.14	0.1262	
Hemiptera	7.54 ± 4.94	1.69 ± 0.83	0.89 ± 0.53	6.50	0.0388	
Other insects^§^	—	1.24 ± 1.13	0.02 ± 0.02	8.17	0.0169	SI > IOP
Plant matter	1.57 ± 1.12	0.11 ± 0.11	—	8.17	0.0169	IOP > E

*Indicates significant differences after sequential Bonferroni correction.

^†^Contains 2 different types/sizes of ants.

^‡^Contains 9 types of unidentified ants.

^§^Contains 2 types of unidentified insect.

We found a total of 3648 prey items in historical gut samples from the museum specimens. There were 18 prey types identified from the historical Isle of Palms samples and 13 from the historical Sullivan’s Island samples. There were more *Pheidole* and *Forelius* in the historical Sullivan’s Island gut samples than in the present-day Sullivan’s Island fecal pellets (Supplementary Table 3). There were more unidentified ants found in the historical gut samples and the present-day fecal samples than in the present-day stomach contents at Isle of Palms (Supplementary Table 4). Overall, ants were the primary prey found, composing 96.69% of the diet by number in the historical Isle of Palms gut contents, 95.73% of historical Sullivan’s Island gut contents, and 95.95% of the present Isle of Palms stomach contents.

**Figure 5:**
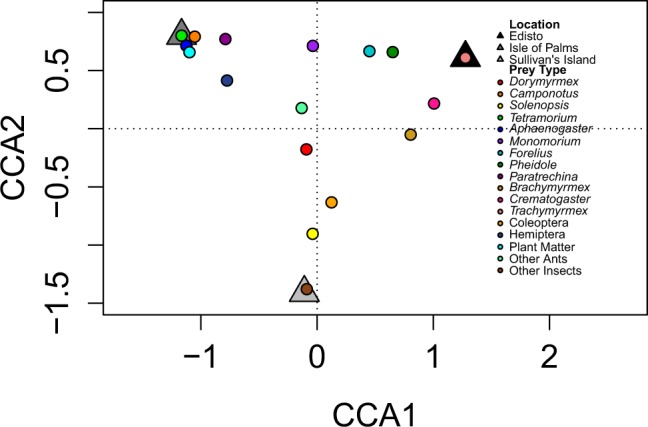
Results of the canonical correspondence analysis for the fecal pellet data. Triangles represent locations and circle represent prey types. Prey types that were commonly consumed at all locations are closer to the center. Prey types that were found exclusively at one site are furthest away from the origin and closest to the location.

Canonical correspondence analysis on fecal pellets showed that a significant amount of the variation in lizard prey was explained by location (F_2,27_ = 2.9556, *P* < 0.001). CCA1 explained 10.25% and CCA2 explained 7.71%, for a total of 17.96% of the variation in prey explained by site (Fig. 5).

## Discussion

Introduction events with a small number of individuals often go through population bottlenecks and experience genetic drift, resulting in an introduced population that has less genetic variation than the source^72,73^. This appears to be the case for SC populations of *Phrynosoma cornutum*. Both the nuclear and mitochondrial genetic data show that the lizards in SC have less variation than areas in the native range, even compared to another barrier island off the coast of Texas which also appears to be bottlenecked. Diversity for both genetic markers was very low throughout the introduced range, suggesting that the founding event was an introduction of a relatively small number of individuals. The results of the microsatellite assignment test and haplotype relationships indicate that SC lizards are predominately from south Texas but also have some northern ancestry, suggesting there was a minimum of two introductions from separate regions in Texas.

Loss of genetic diversity due a major bottleneck can eventually lead to inbreeding depression which can reduce the ability of a population to adapt and lead to population extinction^74–77^. Yet, the low genetic diversity observed for SC *P. cornutum* appears to have had little negative effect over the past ~75 years. The genetic evidence suggests that either Sullivan’s Island or the neighboring Isle of Palms was the original introduction site since these areas have the highest genetic diversity and then individuals were introduced from the Sullivan’s Island/Isle of Palms area to Edisto Beach which has the lowest level of genetic diversity. The populations have all survived major disturbances (example: Hurricane Hugo in 1989), further suggesting that loss of genetic diversity has not impaired population persistence. The surprising part of these results is that this specialized lizard species, even after a severe loss of genetic diversity, has found success after introduction on barrier islands in SC.

There are many other examples of successful establishment of non-native species after severe bottlenecks or extremely small founder events^73,78–81^. But, how do non-native species survive after loss of genetic diversity? First, loss of diversity does not necessarily equate to loss of fitness. For example, loss of genetic diversity in argentine ants (*Linepithema humile*) is related to reduction of aggressive behavior such that colonies from introduced populations do not attack each other and can build super-colonies that overwhelm native ants^73^. We do not know if fitness (fecundity, survivorship) in SC differs from the native range because we lack such data. Second, there might be recovery of genetic variation after the initial bottleneck during a ‘lag phase’^6^. Mechanisms such as purging of deleterious alleles, accumulation of advantageous mutations, or genetic admixture due to repeated introductions can occur during the lag phase^6^. There is evidence for admixture between southern and northern regions in SC populations although this does not appear to have enhanced the genetic diversity of these populations compared to native populations. The role of purging is unclear and mutation seems unlikely given the short time since introduction and very low genetic diversity in SC. Third, empty niches may offer the opportunity for establishment of non-native species, even with low genetic diversity and its associated risks. Wall lizards (*Podaris muralis*) were established in Cincinnati, Ohio, USA from a founding population of ~ 10 individuals and show the genetic signature of a severe bottleneck^79^. This population subsequently expanded throughout the city resulting in additional bottlenecks and yet these populations have persisted. This result was, in part, explained by an open niche that was unoccupied by native lizards^82^.

Dietary analysis revealed that SC *P. cornutum* consume mostly ants (94%) which is not surprising as *P. cornutum* is an ant specialist in the native western range^43,46,47,83^. However, no harvester ants (*Pogonomyrmex* spp.) were found in any of the present day or historical samples from SC and this was unexpected because harvester ants are the main prey of *P. cornutum* in the lizard’s native range (Table 4). The Florida harvester ant (*Pogonomyrmex badius*) does inhabit the SC study area, but they do not appear to be very abundant on the parts of the dunes where we routinely captured *P. cornutum* (EJM unpublished data). During the two summers of sampling, we only saw two harvester ant mounds on Sullivan’s Island and three on Edisto Beach, and lizards were not found in the immediate area of the mounds. It is unclear if the lack of harvester ants in the lizards’ diet is due to lack of available prey or if the introduced lizard has decimated local harvester ant populations. It is worth noting that harvester ants are abundant on the entire dune system at Kiawah Island, SC, a barrier island just north of Edisto and south of Sullivan’s and Isle of Palms (EJM, unpublished data). However, it is clear that introduced *P. cornutum* are persisting on SC barrier islands without eating harvester ants and this represents a shift in resource use.

**Table 4:** Comparison of *P. cornutum* diet from various sources.

Source	Present study	Blackshear & Richerson 1999	Davis 1941	Eifler *et al*. 2012	Endriss 2006	Pianka & Parker 1975	Whitford & Bryant 1979
Location	SC	TX, NM, Mexico	MI	AZ	OK	N/A	NM
Sample size	46	30	1	25	3	351	34
**Ants**							
Aphaenogaster	5.95						
Brachymyrmex	4.21						
Camponotus	0.89						
Crematogaster	1.61	0.69			25.86		
Dorymyrmex	51.42	6.17					
Forelius	7.45						
Formica		15.74	10.00		23.28		
Leptothorax			15.00				
Monomorium	0.12				10.34		
Myrmecocystus		0.01					
Neivamyrmex		20.09					
Novomessor		1.37					
Paratrechina	1.08				1.72		
Pheidole	12.49	1.96			9.48		
Pogonomyrmex		52.00		97.69			88.73
Prenolepis			51.67				
Pseudomyrmex		0.01					
Solenopsis	4.54	1.86					
Tapinoma					4.31		
Tetramorium	3.59						
Trachymyrmex	0.01						
Veromessor		0.07					
Unknown	0.87				12.93		
**Total Ants**	**94.23**	**100.00**	**76.67**	**99.95**	**87.93**	**69.00**	**100.00**
**Insects**							
Coleoptera	1.91		6.67	0.05	6.03		
Diptera	0.22						
Hemiptera	2.54		5.00		1.72		
Hymenoptera	0.01		1.67		0.86		
Lepidoptera	0.01						
Orthoptera			8.33				
Unknown	0.27						
**Other**							
Araneae	0.33		1.67		0.86		
Isopoda					2.59		
Plant Matter	0.49						

Ants are classified by genus and other insects are classified by order based on the current taxonomy. Values reported are percent eaten by number.

In the absence of harvester ants, *P. cornutum* living in SC have shifted their diet to prey that are available. While we do not have data on the insect abundance on the dunes, the most common prey eaten (*Dorymyrmex*) were also the most commonly observed insect on the dunes (EJM unpublished data). Previous studies have found that *P. cornutum* will consume other ants and insects in the absence of harvester ants^84–86^. A study on the desert horned lizard (*Phrynosoma platyrhinos*) suggests that horned lizards are opportunistic feeders that consume ants based on availability and size instead of specifically preferring harvester ants^87^. Similarly, the coast horned lizard (*Phrynosoma coronatum*) has been known to shift its diet when harvester ants become limited. In southern California, the invasive Argentine ant (*Linepithema humile*) has displaced the native harvester ants, and the lizards shifted to eating insects and other smaller ants in these areas^88,89^. Interestingly, *Dorymyrmex insanus* was the ant most commonly eaten by *P. coronatum* when harvester ants were absent^88^. These results may suggest that *Phrynosoma* species have greater dietary flexibility than is commonly thought and can persist without harvester ants given that suitable substitute ant species are present.

*Dorymyrmex* ants may be such a species. *Dorymyrmex* colonies are found in open, dry environments with many species distributed across the U.S. and several species occupying sand dune habitats in the southeast^51^. *Dorymyrmex* ants are very active with workers foraging during the day^51^. The activity pattern of *Dorymyrmex* coincides with *P. cornutum* activity and these ants are abundant, making *Dorymyrmex* a suitable prey item for the lizards. These results suggest that *P. cornutum* may be opportunistic myrmecophages and that they do not require harvester ants as the main component or even as part of their diet.

Two other myrmecophages are commonly found on or near sand dunes in SC: eastern narrow-mouth toads (*Gastrophryne carolinensis*) and antlions (Neuroptera, Myrmeleontodae). Narrow-mouth toads are nocturnal and primarily consume nocturnally active ants^90^. They are found in the swales between dune ridges and in maritime forests adjacent to the dunes (EJM pers. obs). Narrow-mouth toads and horned lizards likely have little dietary overlap because *Phrynosoma cornutum* is diurnal and is found on the dune ridges and drier soils. Antlions (Neuroptera, Myrmeleontodae) are abundant on the sand dunes and can have significant effects on ant ecology and behavior^91^. Antlion species compete both intra- and interspecifically^92,93^. Thus, it is likely that antlions and horned lizards compete for the same ants. However, given the success of *P. cornutum* at establishing populations on at least three barrier islands, it seems unlikely that antlion distributions could limit horned lizard establishment success. Other dune dwelling lizard species (*Aspidoscelis sexlineata*, *Plestiodon* spp., *Ophisaurus* spp.) include relatively low percentages of ants in their diets^94–96^. The lack of ant predators coupled with an abundance of ants and the coarse similarity in structural habitat to Western US deserts and grasslands may prime barrier island sand dune communities for establishment of ant-eating desert lizards, such as horned lizards. Thus, the invasion success of a dietary specialist may depend on an appropriate resource pool with little competition from native species, i.e. an open niche.

The morphological differentiation of the three SC populations may be evidence of local adaptation, a plastic response to local selection or random drift due to founding effects. Other studies on phrynosomatid lizards have found that morphology is correlated with microhabitat use^56,97^ and environmental conditions^57^. Adult females and males from Sullivan’s Island have the longest limbs for their body size and this trait is correlated with running ability and sprint speed in phrynosomatid lizards^98^. This may be important on Sullivan’s Island because this is the only site that has an established population of coyotes (*Canis latrans* Say, 1823) and where domestic dogs (*Canis familiaris* Linnaeus, 1758) frequently run off leash, both of which are known predators of horned lizards^99^. Additionally, diet differed across the three SC populations suggesting that horned lizards are adjusting their dietary preferences to local prey, which may exert selection on limb and head morphology^100^. However, genetic drift may also be driving population level morphological divergence in SC, which is a possibility given small populations founded from a few individuals.

We found significant differences in body size and shape between the SC and Texas populations. Although the data are limited, it is clear that females from South Texas are morphologically distinct from all SC populations and that both males and females from West Texas differ from SC populations. Studies of additional Texas populations are needed to better link population level genetic variation to morphological differentiation. Measurements of habitat use, predation pressure and climate would be useful as population-level differences in these variables might indicate different selective regimes. Additionally, a common garden study among populations (both SC and TX) could differentiate plasticity vs. adaptation as the cause of population level morphological differentiation.

## Supplementary information

Supplementary Information

## Data Availability

Data are availabe at Figshare. [10.6084/m9.figshare.9170765.v1].
