# Supplementary material for: Genetics, morphology and diet of introduced populations of the ant-eating Texas Horned Lizard (Phrynosoma cornutum)
Source: Sci Rep. 2019 Aug 7;9:11470. doi: 10.1038/s41598-019-47856-4 (PMC6685972; doi:10.1038/s41598-019-47856-4)
Supplement: Supplementary file 1 — Supplementary Information [file 41598_2019_47856_MOESM1_ESM.docx]

Genetics, morphology and diet of introduced populations of the ant-eating Texas Horned Lizard (*Phrynosoma cornutum*)

Courtney Heuring^1^, Diane Barber^2^, Nathan Rains^3^, Devin Erxleben^3^, Cameron Martin^3^, Dean Williams^4^, Eric J McElroy^1*^

^1^Department of Biology, College of Charleston, Charleston, South Carolina, USA, 29412

^2^Fort Worth Zoo, Fort Worth, Texas, USA, 76110

^3^Texas Parks and Wildlife Department, Austin, Texas, USA, 78744

^4^Department of Biology, Texas Christian University, Fort Worth, Texas, USA, 76129

*corresponding author: [mcelroye@cofc.edu](mailto:mcelroye@cofc.edu)

Supplementary Information

**Supplementary Figure 1.** Species accumulation curves and the proportion of total prey represented by those species for fecal pellet samples from Isle of Palms (A & B), Sullivan’s Island (C & D), and Edisto (E & F). N = 10 fecal pellets per site.

**Supplementary Figure 2.** A) Species accumulation curve and B) the proportion of total prey represented by those species for stomach contents (N = 9) from Isle of Palms *P.* *cornutum.*

**Supplementary Table 1.** Sample sizes for morphological measurements (number of individuals).

| **Location** | **Sex/Age** | **Sample Size (N)** |
| --- | --- | --- |
| Edisto | female | 9 |
| Edisto | male | 6 |
| Edisto | juvenile | 10 |
| Isle of Palms | female | 19 |
| Isle of Palms | male | 20 |
| Isle of Palms | juvenile | 18 |
| Sullivan’s Island | female | 18 |
| Sullivan’s Island | male | 10 |
| Sullivan’s Island | juvenile | 8 |
| South Texas | female | 5 |
| West Texas | female | 7 |
| West Texas | male | 14 |

**Supplementary Table 2.** Genetic diversity at 10 microsatellite loci for three introduced *P. cornutum* populations in SC. N – number of individuals, N_A_ – number of alleles, A_R_ is allelic richness standardized for 17 individuals, H_O_ – observed heterozygosity, H_E_ – expected heterozygosity, F – inbreeding coefficient, * indicates evidence for null alleles as determined by MICRO-CHECKER

| **Location** | **Locus** | **N** | **N_A_** | **A_R_** | **H_O_** | **H_E_** | **F** |
| --- | --- | --- | --- | --- | --- | --- | --- |
| Isle of Palms | Pc41 | 37 | 4 | 4.0 | 0.76 | 0.69 | -0.11 |
|  | PcD01 | 37 | 5 | 4.5 | 0.32 | 0.59 | 0.44* |
|  | PcD09 | 37 | 5 | 4.6 | 0.46 | 0.66 | 0.29* |
|  | Pc70 | 37 | 4 | 3.9 | 0.51 | 0.54 | 0.03 |
|  | PcD14 | 37 | 5 | 4.5 | 0.59 | 0.55 | -0.09 |
|  | PcD20 | 37 | 3 | 2.9 | 0.46 | 0.42 | -0.11 |
|  | PcD52 | 37 | 3 | 3.0 | 0.62 | 0.61 | -0.03 |
|  | Pc83 | 37 | 4 | 3.9 | 0.59 | 0.64 | 0.06 |
|  | PcD26 | 37 | 4 | 3.9 | 0.68 | 0.70 | 0.02 |
|  | PcD53 | 37 | 4 | 4.0 | 0.81 | 0.74 | -0.11 |
| Sullivan's Island | Pc41 | 29 | 4 | 4.0 | 0.52 | 0.50 | -0.06 |
|  | PcD01 | 28 | 5 | 4.8 | 0.50 | 0.66 | 0.22 |
|  | PcD09 | 27 | 4 | 4.0 | 0.74 | 0.73 | -0.04 |
|  | Pc70 | 29 | 3 | 3.0 | 0.45 | 0.41 | -0.11 |
|  | PcD14 | 29 | 2 | 2.0 | 0.72 | 0.51 | -0.45 |
|  | PcD20 | 29 | 3 | 2.8 | 0.24 | 0.27 | 0.10 |
|  | PcD52 | 29 | 2 | 2.0 | 0.38 | 0.35 | -0.09 |
|  | Pc83 | 27 | 3 | 3.0 | 0.59 | 0.58 | -0.04 |
|  | PcD26 | 29 | 4 | 4.0 | 0.72 | 0.69 | -0.07 |
|  | PcD53 | 29 | 3 | 3.0 | 0.45 | 0.47 | 0.03 |
| Edisto Beach | Pc41 | 25 | 4 | 3.8 | 0.08 | 0.12 | 0.31 |
|  | PcD01 | 24 | 2 | 2.0 | 0.29 | 0.36 | 0.17 |
|  | PcD09 | 23 | 4 | 4.0 | 0.70 | 0.68 | -0.05 |
|  | Pc70 | 25 | 3 | 2.9 | 0.52 | 0.51 | -0.03 |
|  | PcD14 | 25 | 4 | 3.9 | 0.72 | 0.69 | -0.07 |
|  | PcD20 | 25 | 3 | 3.0 | 0.32 | 0.29 | -0.14 |
|  | PcD52 | 25 | 3 | 2.9 | 0.20 | 0.33 | 0.39 |
|  | Pc83 | 23 | 2 | 2.0 | 0.17 | 0.16 | -0.10 |
|  | PcD26 | 23 | 3 | 3.0 | 0.61 | 0.57 | -0.09 |
|  | PcD53 | 23 | 3 | 3.0 | 0.52 | 0.49 | -0.09 |

**Supplementary Table 3.** Diet composition and Kruskal-Wallis results for historical Sullivan's Island gut contents and present-day Sullivan’s Island fecal pellets. Means ± SE are reported.

|  | Diet Composition (%) | |  | Kruskal-Wallis Results | | |  |
| --- | --- | --- | --- | --- | --- | --- | --- |
|  | Historical SI n = 3 | Present SI n = 10 |  | *H* |  | *P*-value |  |
| Prey type |  |  |  |  |  |  | Group comparisons |
| *Dorymyrmex* | 54.67 ± 21.97 | 75.01 ± 5.98 |  | 0.46 |  | 0.4990 |  |
| *Solenopsis* | 0.53 ± 0.53 | 8.53 ± 2.72 |  | 6.45 |  | 0.0111 | Present > Historical |
| *Tetramorium* | 0.06 ± 0.06 | — |  | 3.33 |  | 0.0679 |  |
| *Aphaenogaster* | — | 0.51 ± 0.51 |  | 0.30 |  | 0.5839 |  |
| *Forelius* | 13.30 ± 12.12 | 0.04 ± 0.04 |  | 8.35 |  | 0.0038* | Historical > Present |
| *Pheidole*† | 25.65 ± 24.04 | — |  | 11.76 |  | 0.0006* | Historical > Present |
| *Paratrechina* | 0.86 ± 0.79 | — |  | 7.22 |  | 0.0072 | Historical > Present |
| *Brachymyrmex* | — | 6.11 ± 4.07 |  | 4.35 |  | 0.0369 | Present > Historical |
| *Crematogaster* | — | 1.44 ± 1.44 |  | 0.30 |  | 0.5839 |  |
| Other ants‡ | 0.67 ± 0.54 | 0.79 ± 0.58 |  | 0.75 |  | 0.3858 |  |
| Coleoptera | 1.41 ± 0.89 | 4.53 ± 2.83 |  | 0.26 |  | 0.6121 |  |
| Hemiptera | 1.47 ± 0.85 | 1.69 ± 0.83 |  | 0.72 |  | 0.3974 |  |
| Other insects§ | — | 1.24 ± 1.13 |  | 2.09 |  | 0.1483 |  |
| Plant matter | 1.39 ± 0.92 | 0.11 ± 0.11 |  | 3.78 |  | 0.0520 |  |

* indicates significant differences after sequential Bonferroni correction

† contains 2 different types/sizes of ants

‡ contains 5 types of unidentified ants

§ contains 1 type of unidentified insect

**Supplementary Table 4.** Diet composition and Kruskal-Wallis test results for historical Isle of Palms gut contents, present-day Isle of Palms stomach contents, and present-day Isle of Palms fecal pellets. Means ± SE are reported.

|  | Diet Composition (%) | | |  | Kruskal-Wallis Results | | |  |
| --- | --- | --- | --- | --- | --- | --- | --- | --- |
|  | Historical IOP gut n = 4 | Present IOP stomach n = 9 | Present IOP fecal n = 10 |  | *H* |  | *P*-value |  |
| Prey type |  |  |  |  |  |  |  | Group comparisons |
| *Dorymyrmex* | 29.87 ± 10.29 | 33.01 ± 11.11 | 57.43 ± 9.55 |  | 3.23 |  | 0.1984 |  |
| *Camponotus* | 0.42 ± 0.42 | 3.03 ± 1.48 | 1.13 ± 0.80 |  | 1.63 |  | 0.4432 |  |
| *Solenopsis* | 17.03 ± 16.73 | 2.96 ± 1.18 | 1.20 ± 0.34 |  | 0.59 |  | 0.7434 |  |
| *Tetramorium* | 0.04 ± 0.04 | 13.41 ± 9.54 | 4.43 ± 2.03 |  | 1.36 |  | 0.5077 |  |
| *Aphaenogaster* | 0.22 ± 0.22 | 15.67 ± 9.76 | 12.65 ± 4.60 |  | 3.94 |  | 0.1394 |  |
| *Monomorium* | 0.12 ± 0.08 | 0.07 ± 0.05 | 0.23 ± 0.13 |  | 0.93 |  | 0.6273 |  |
| *Forelius* | 12.50 ± 10.43 | 17.00 ± 6.50 | 3.36 ± 1.54 |  | 0.58 |  | 0.7470 |  |
| *Pheidole*† | 31.75 ± 17.53 | 6.15 ± 4.19 | 8.08 ± 5.37 |  | 5.26 |  | 0.0721 |  |
| *Paratrechina* | 2.97 ± 1.82 | 3.83 ± 3.68 | 0.07 ± 0.07 |  | 6.89 |  | 0.0319 | Gut > Fecal |
| *Brachymyrmex* | — | 0.82 ± 0.82 | 0.13 ± 0.10 |  | 0.89 |  | 0.6414 |  |
| Other ants‡ | 1.78 ± 0.84 | — | 1.34 ± 0.54 |  | 13.76 |  | 0.0010* | Gut > Stomach , Fecal > Stomach |
| Coleoptera | 1.36 ± 0.50 | 0.65 ± 0.40 | 0.84 ± 0.20 |  | 3.68 |  | 0.1590 |  |
| Hemiptera | 1.71 ± 0.79 | 0.48 ± 0.19 | 7.54 ± 4.94 |  | 7.56 |  | 0.0228 | Fecal > Stomach |
| Other insects§ | — | 2.87 ± 1.79 | — |  | 9.33 |  | 0.0094 | Stomach > Fecal |
| Plant matter | 0.23 ± 0.20 | 0.06 ± 0.04 | 1.57 ± 1.12 |  | 2.88 |  | 0.2368 |  |

* indicates significant differences after sequential Bonferroni correction

† contains 2 different types/sizes of ants

‡ contains 8 types of unidentified ants

§ contains 4 types of non-ant insects (Araneae, Hymenoptera, Lepidoptera, Diptera)
